# Addition of binahong (*Anredera cordifolia*) leaf powder to diets to produce eggs with low cholesterol

**DOI:** 10.14202/vetworld.2020.604-608

**Published:** 2020-03-31

**Authors:** Sri Kismiati, Hanny Indrat Wahyuni, Rina Muryani, Dwi Sunarti, Sri Sumarsih

**Affiliations:** Department of Animal Science, Faculty of Animal and Agricultural Science, Diponegoro University, Tembalang Campus, Semarang, Central of Java, Indonesia

**Keywords:** binahong, cholesterol, high-density lipoprotein, low-density lipoprotein, triglyceride

## Abstract

**Aim:**

The aim of this study was to evaluate the effect of the addition of binahong leaf powder to quail rations on the production and quality of eggs.

**Materials and Methods:**

The study involved the use of two hundred 7-week-old quails housed evenly in 20 wire cages with a body weight of 123.77±0.72 g. The quails were treated as follows: Ration without binahong leaf powder (T0), addition 2% of binahong leaf powder (T1), addition 4% of binahong leaf powder (T2), and addition 6% of binahong leaf powder (T3). The study used a completely randomized design. The parameters measured were the production, weight, and characteristics of the eggs, as well as the cholesterol, triglyceride, high-density lipoprotein (HDL), low-density lipoprotein (LDL), and egg protein content in the yolk.

**Results:**

The addition of 2-6% binahong powder did not significantly affect egg production, egg characteristics, or egg protein content, but significantly (p<0.05) affected the cholesterol, triglyceride, HDL, and LDL contents in yolk. The cholesterol, triglyceride, and LDL contents decreased significantly in T1, whereas HDL increased significantly in T2 and T3.

**Conclusion:**

The addition of 2% binahong was enough to obtain healthy quail eggs with low levels of cholesterol, triglyceride, and LDL.

## Introduction

Quail egg contains many high-quality nutrients but also high cholesterol [1]. The protein content in a quail egg is approximately 13.30% [1]. The cholesterol content in a quail egg is higher than in a chicken egg. The cholesterol content in the yolk of a quail egg is 6.79 mg/dl, whereas the cholesterol content in chicken egg yolk is 4.03 mg/dl [2]. Overconsumption of cholesterol increases the blood cholesterol level, which leads to heart disease; thus, some people are afraid of consuming quail egg. Catapano and Wiklund [3] stated that blood cholesterol, particularly low-density lipoprotein (LDL) cholesterol, has a positive correlation with the occurrence of atherosclerosis. The consumption of high-density lipoprotein (HDL) derived from quail egg increases the blood serum HDL level and decreases atherosclerotic plaques in rabbits. Thus, egg HDL may be used as an anti-atherosclerotic agent for patients with cardiovascular disease [4]. An attempt to obtain quail egg with low cholesterol and high HDL contents needs to be carried out.

Binahong (*Anredera cordifolia*) is a wild plant with a rapid growth rate that requires no complicated cultivation; thus, binahong is abundant [5]. Sutrisno *et al*. [5] and Leliqia *et al*. [6] revealed that the leaf of binahong contains bioactive compounds, such as flavonoids, tannins, saponins, phenols, and steroids, and Astuti *et al*. [7] reported that the leaf also contains terpenoid, which may potentially increase pancreatic insulin secretion. Furthermore, Hasbullah [8] documented that binahong leaf exhibits hypolipidemic properties. Kamboh *et al*. [9] showed that supplementation with a bioflavonoid increases antioxidant and enzyme activities and decreases total cholesterol and triglyceride levels in the serum and breast meat of broiler. A study by Ouyang *et al*. [10] showed that supplementation with 15 mg/kg of alfalfa flavonoid increases HDL levels and decreases the levels of cholesterol, triglyceride, and serum LDL and the percentage of abdominal fat of broiler. Feeding saponin decreases cholesterol, insulin, and blood triglyceride and increases blood HDL synthesis. Saponin also decreases the contents of cholesterol [11] and protein digestibility [12] of broiler meat.

Based on the facts that binahong contain flavonoids, tannins, saponins, phenols, and steroids and it has potentially reduced cholesterol, triglyceride, LDL serum and increased HDL. Hopefully, it will produce a healthy egg product. Therefore, the aim of this study was to evaluate the effect of the addition of binahong leaf powder to rations on the contents of cholesterol, triglyceride, HDL, LDL, and protein in quail eggs.

## Materials and Methods

### Ethical approval

The procedure of using quail in this study has been approved by the Animal Ethics Committee in the Faculty of Animal Sciences, Diponegoro University, Semarang, Indonesia.

### Animals

The study used two hundred 7-week-old female quails with an average body weight of 123.77±0.72 g. The quails were housed in 20 wire cages. Each cage was 90 × 35 × 25 cm and housed 10 quails. The feed consisted of yellow corn, rice bran, pollard, poultry meat meal, soybean meal, CaCO_3_, salt, premix, and binahong leaf powder.

### Experimental design

This study was arranged according to a completely randomized design with four treatments and five repetitions (10 quails per group per repetition). The treatment groups included T0, control ration (without binahong leaf powder); T1, control ration + 2% binahong leaf powder; T2, control ration + 4% binahong leaf powder; and T3, control ration + 6% binahong leaf powder in ration. The composition and nutrient content of the ration are presented in Tables-1 and 2.

**Table-1:** Feed composition of the control ration.

Feed ingredients	%
Yellow corn	48.00
Rice bran	6.00
Pollard	16.00
Poultry meat meal	14.00
Soybean meal	10.00
CaCO_3_	5.50
Salt	0.25
Premix	0.25
Total	100.00

**Table-2:** Nutrition content of the ration.

Nutrient contents	Addition of binahong leaf powder			

	T0 (0%)	T1 (2%)	T2 (4%)	T3 (6%)
Energy metabolic (kkal/kg)	2763.20	2694.12	2628.26	2565.40
Protein (%)	19.77	19.35	18.95	18.56
Fat (%)	4.66	4.58	4.51	4.43
Crude fiber (%)	4.29	4.95	5.56	6.12
Ca (%)	3.15	3.06	2.97	2.88
P (%)	0.79	0.76	0.74	0.71
Lysine (%)	0.96	0.93	0.89	0.86
Methionine (%)	0.48	0.46	0.44	0.42

### Tests and procedures

The recording of egg production was carried out every day during the study and the formula: Production = (number of eggs/number of quails) × 100% was used. Egg weight and egg characteristics were measured every 3 consecutive days weekly. The cholesterol, triglyceride, HDL, LDL, and protein contents in the egg yolk were collected by sampling one egg per experimental unit at the end of the study. The analyses of cholesterol and triglyceride contents were conducted based on the cholesterol p-aminophenazone method [13], HDL and LDL analysis was based on the enzymatic colorimetric method [13], and protein analysis was based on the Kjeldahl method [14].

### Statistical analysis

Data were analyzed as a completely randomized design using one-way analysis of variance and Duncan’s Multiple Range Test.

## Results and Discussion

### Effect of the addition of binahong leaf powder on egg production

The addition of 2-6% of binahong leaf powder, rich in flavonoids and saponins, did not significantly influence quail egg production at 8-14 weeks of age (Table-3). This result was similar to that reported by Kusumanti and Murwani [15], which showed that supplementation of binahong leaf powder does not significantly affect the egg production of laying hens. In line with this, Iskender *et al*. [16] showed that flavonoids have no significant effect on egg production. In contrast, supplementation with the quercetin flavonoid [17] and saponins derived from karaya increases the egg production of laying hens [18]. These inconsistencies may be caused by the differences in the levels of flavonoids and saponins used in the study, as well as the conditions of the study.

**Table-3:** Egg production on addition of binahong leaf powder in quail rations.

Age (weeks)	Addition of binahong leaf powder				p-value

	T0 (0%)	T1 (2%)	T2 (4%)	T3 (6%)	
8	29.71±2.74	28.00±2.166	28.85±2.74	28.57±2.67	0.49
9	39.71±2.34	42.28±1.91	41.42±2.67	41.14±2.55	0.53
10	45.14±4.58	46.09±1.47	47.23±2.34	45.14±3.08	0.57
11	46.64±2.42	49.49±1.38	48.73±3.83	47.63±1.73	0.59
12	56.38±2.49	52.34±1.86	54.50±1.37	54.57±1.76	0.67
14	56.17±2.47	57.55±1.45	57.73±1.85	58.74±1.93	0.69

### Effect of binahong leaf powder on egg characteristics

The addition of binahong leaf powder did not significantly affect the characteristics of the egg, including egg weight, yolk weight, albumen weight, and eggshell weight (Table-4). It has been hypothesized that the bioactive component of binahong leaf may positively affect the egg characteristics. However, this study revealed different results. According to Leke *et al*. [19], flavonoids of papaya seeds increase egg quality (egg yolk, albumen, and eggshell weight) of Indonesian hens. Moreover, Afrose *et al*. [18] reported that supplementation with 25, 50, and 75 mg/kg of karaya saponin increases egg weight, yolk weight, and albumen weight of laying hens, whereas Ayasan *et al*. [20] reported that supplementation of *Yucca schidigera* powder, with 120 ppm of saponin, increases egg weight but does not affect the eggshell weight. As in our study, one study showed that supplementation with 0.2-0.6 g/kg of flavonoid does not significantly influence egg quality [17]. In addition, supplementation with flavonoids does not significantly influence eggshell weight, as reported by Iskender *et al*. [16]. The differences in the nature and levels of bioactive compounds, the nutritional values of rations, and the conditions of study may be responsible for these divergent results.

**Table-4:** Influence of addition of binahong leaf powder on quail eggs characteristic.

Parameters	Addition of binahong leaf powder				p-value

	T0 (0%)	T1 (2%)	T2(4%)	T3 (6%)	
Egg weight (g)	9.99±0.29	10.01±0.11	10.03±0.11	10.03±0.27	0.38
Yolk weight (g)	3.65±0.13	3.92±0.25	3.68±0.12	3.93±0.17	0.57
Albumen weight (g)	4.68±0.29	4.53±0.18	4.47±0.25	4.60±0.19	0.53
Eggshell weight (g)	1.14±0.28	1.12±0.15	1.14±0.17	1.01±0.10	0.32
Yolk weight (%)	38.77±2.34	41.80±2.72	40.01±1.39	41.13±0.79	0.51
Albumen weight (%)	49.63±2.78	48.27±2.07	48.56±2.70	48.13±1.58	0.73
Eggshell weight (%)	11.43±2.73	11.07±1.60	11.41±1.73	10.11±1.14	0.12

### Cholesterol, triglyceride, HDL, LDL, and protein contents in yolk

The data on cholesterol, triglyceride, HDL, LDL, and protein contents in yolk after binahong leaf powder supplementation are shown in Table-5. The addition of 2% of binahong leaf powder decreased the level of cholesterol in the yolk of quail eggs (p<0.05). Flavonoids and saponins in the binahong leaf powder may inhibit cholesterol absorption; hence, cholesterol deposition may be reduced [21-23]. This is supported by Lien *et al*. [24] who showed that the consumption of flavonoids increases the excretion of cholesterol. In addition, flavonoids may increase reverse cholesterol transport (RCT), resulting in high cholesterol excretion; thus, there will be a lower cholesterol level in yolk. However, 4 and 6% binahong leaf powder did not affect the cholesterol level.

**Table-5:** Influence of the addition of binahong leaf powder on cholesterol, triglyceride, HDL, LDL, and protein of quail egg yolk.

Parameters	Addition of binahong leaf powder				p-value

	T0 (0%)	T1 (2%)	T2 (4%)	T3 (6%)	
Yolk cholesterol (mg/g)	56.15±5.04^a^	23.77±3.75^b^	51.97±4.63^a^	51.35±7.55^a^	<0.01
Yolk triglyceride (mg/g)	903.22±58.21^a^	608.62±22.46^c^	778.34±53.59^b^	811.63±104.01^b^	<0.01
Yolk HDL (mg/g)	36.86±1.32^b^	34.8b±1.58^b^	48.62±1.67^a^	47.52±1.58^a^	<0.01
Yolk LDL (mg/g)	29.18 ±1.59^ a^	16.06b±0.33^b^	17.09±0.37^b^	16.28±0.36^b^	<0.01
Protein of yolk (%)	30.16±2.78	30.37±.82	29.15±0.81	30.19±2.55	0.00

Mean within a row for each parameter with different superscripts are significantly different (p<0.01)

The addition of binahong leaf powder decreased the triglyceride content in quail yolk (Table-5). As shown in our study, supplementation with a flavonoid extracted from the root of *Scutellaria baicalensis* Georgi decreases triglyceride content in the serum of broiler [25]. Likewise, Kamboh and Zhu [21] and Ouyang *et al*. [10] reported that the consumption of flavonoids decreases the level of triglyceride in the serum and meat of broiler. According to Hsu and Yen [26] and Nagai *et al*. [27], flavonoids may inhibit intracellular triglyceride synthesis in the liver, which results in lower triglyceride deposition in quail egg yolk. In addition, flavonoid content in binahong leaf powder may increase the expression of peroxisome proliferator-activated receptor α in the liver, which is involved in lipid metabolism, especially fatty acid oxidation [10,28].

The content of yolk HDL increased with the increased concentration of binahong leaf powder in the quail rations (p<0.05). Similar to the result of our study, Afrose *et al*. [29] documented that saponins increase HDL content in broiler meat. Supplementation with karaya saponin also increases HDL content in quail egg [30]. Furthermore, Smith *et al*. [31] stated that supplementation with saponin extract increases the HDL content in the blood, liver, kidneys, and heart tissues of white mice. Kamboh and Zhu [21] reported that flavonoids increase the HDL content in serum and HDL deposition in the breast meat of broiler. The mechanism by which binahong leaf increased HDL concentration in quail egg yolk is not known; however, the role of binahong leaf powder in the RCT mechanism seems to be attributable to the increased HDL content in quail yolk. Millar *et al*. [23] and Marques *et al*. [32] reported that the increase in the RCT mechanism due to binahong leaf powder supplementation is accompanied by an increase in the level of HDL. Thus, this mechanism seems to be related to the contribution of HDL on RCT.

Treatment with binahong leaf powder decreased the level of LDL in the quail egg yolk (Table-5). Afrose *et al*. [30] reported that saponins decrease the LDL content of quail egg. Likewise, Chaudhary *et al*. [33] stated that saponins potentially decrease the LDL content of meat. In addition, supplementation with saponin extract decreases LDL content in the blood, liver, kidneys, and heart tissues of white mice [31]. Flavonoids have been reported to decrease the LDL level in the blood of broiler [10,21,25]. Furthermore, Zhou *et al*. [25] stated that flavonoid supplementation also decreases the LDL content of the breast meat of broiler. Binahong leaf extract contains saponins, which bind bile acid and form large mixed micelles. Cholesterol in the micelles cannot be absorbed by microvilli on the surface of intestinal epithelial cells, which causes a decrease in total and LDL cholesterol levels [34]. Saponins inhibit fat metabolism through the inhibition of lipase secretion and decrease cholesterol and LDL [35], but increase HDL [36], contents in the blood. Flavonoids are active compounds that reduce the activity of fatty acid synthase and reduce the level of LDL in animal tissues [10].

The addition of 2-6% binahong leaf powder did not significantly influence the protein content of egg yolk. This contrasted with a previous study. Iskender *et al*. [16] reported that supplementation with flavonoids (hesperidin, naringin, and quercetin) increases egg protein. Ahmad *et al*. [37] documented that bioactive compounds from *Moringa oleifera* leaf extracts increase the protein content in yolk.

In general, the protein content in an egg is derived from the feed. In this study, the inclusion of binahong leaf powder was accompanied by the decreased crude protein in the ration. Hence, the protein-increasing effect by binahong leaf powder seemed to be inhibited by lower protein content in the quail ration due to the binahong leaf inclusion.

## Conclusion

The addition of 2% binahong leaf powder is best for lowering cholesterol, triglyceride, and LDL levels in quail egg.

## Authors’ Contributions

SK conducted the research, data collection, and drafting of the article. HIW developed the feeding concept and supervised the research, RM advised the experimental design, DS conducted data analysis, and SS performed laboratory analysis. All authors read and approved the final manuscript.
